# Development and Validation of an Instrument for Measuring Attitudes and Beliefs about Complementary and Alternative Medicine (CAM) Use among Cancer Patients

**DOI:** 10.1155/2012/798098

**Published:** 2012-05-30

**Authors:** Jun J. Mao, Steve C. Palmer, Krupali Desai, Susan Q. Li, Katrina Armstrong, Sharon X. Xie

**Affiliations:** ^1^Department of Family Medicine and Community Health, University of Pennsylvania Health System, Philadelphia, PA 19104, USA; ^2^Center for Clinical Epidemiology and Biostatistics, University of Pennsylvania Health System, Philadelphia, PA 19104, USA; ^3^Abramson Cancer Center, University of Pennsylvania Health System, Philadelphia, PA 19104, USA; ^4^Department of Psychiatry, University of Pennsylvania Health System, Philadelphia, PA 19104, USA; ^5^Department of Medicine, University of Pennsylvania Health System, Philadelphia, PA 19104, USA

## Abstract

Despite cancer patients' extensive use of complementary and alternative medicine (CAM), validated instruments to measure attitudes, and beliefs predictive of CAM use are lacking. We aimed at developing and validating an instrument, attitudes and beliefs about CAM (ABCAM). The 15-item instrument was developed using the theory of planned behavior (TPB) as a framework. The literature review, qualitative interviews, expert content review, and cognitive interviews were used to develop the instrument, which was then administered to 317 outpatient oncology patients. The ABCAM was best represented as a 3-factor structure: expected benefits, perceived barriers, and subjective norms related to CAM use by cancer patients. These domains had Eigenvalues of 4.79, 2.37, and 1.43, and together explained over 57.2% of the variance. The 4-item expected benefits, 7-item perceived barriers, and 4-item subjective norms domain scores, each had an acceptable internal consistency (Cronbach's alpha) of 0.91, 0.76, and 0.75, respectively. As expected, CAM users had higher expected benefits, lower perceived barriers, and more positive subjective norms (all *P* < 0.001) than those who did not use CAM. Our study provides the initial evidence that the ABCAM instrument produced reliable and valid scores that measured attitudes and beliefs related to CAM use among cancer patients.

## 1. Introduction

The use of complementary and alternative medicine (CAM) is extensive among cancer patients [1–3]. Many cancer patients turn to CAM therapies in addition to their conventional treatments to deal with ongoing health issues and increased symptom burden such as recurring pain and psychological distress [4–6]. Population-based studies have demonstrated that cancer patients are more likely to use CAM than the general population [7, 8]; thus, it is important to understand the attitudes and beliefs related to CAM use among cancer patients in order to create a more personalized integrative health system to tailor therapies to individual beliefs and decision factors [9].

Why individuals use CAM is complex, personal, and driven by multiple factors. Sociodemographic factors such as female sex, younger age, higher education and income, and white race have been associated with CAM use in epidemiology studies [1, 10–17]. Research has also found that individuals use CAM to improve their physical and emotional health, enhance quality of life, strengthen the immune system, minimize the side effects of conventional medical treatments, and exert positive effects on cancer [11, 12, 18–22]. Other psychological or culture factors relate to CAM use may include being open to new experiences, preferring natural/holistic approaches to treatment, and the desire to exert a sense of personal control over their illness [13, 20, 23, 24].

Several qualitative studies have provided unique insight into the decision making process utilized by individuals regarding CAM use. Verhoef and White interviewed 31 individuals who used CAM instead of conventional cancer treatments and found several themes related to this decision making process: the family's/friend's experiences with conventional cancer treatment, their own experience with cancer care and physician communication, and patients' beliefs and need for control [25]. Balneaves et al. interviewed breast cancer patients about CAM use and developed the “bridging the gap” model in categorizing individuals into three distinct decision making style: taking one step at a time, playing it safe, and bringing it all together [26]. These kinds of distinctive pathways have also been explored by Caspi et al. among patients with chronic rheumatological disorders [27].

Despite this emerging data, few studies have used a theory-driven, well-developed instrument to guide the inquiry into why individuals use CAM, especially in cancer patients. In a recent systematic review of the research using theoretical models to understand why individuals use CAM, Lorenc et al. found that only 22 studies used a theoretical model to predict CAM use, the majority of which were among noncancer populations [28]. The most commonly used model was the health care utilization model, Andersen's sociobehavioral model [28, 29]. Existing research based on this model predominantly evaluates social demographic factors and symptoms without incorporating a comprehensive assessment of facilitators, barriers, and behavioral predictors of CAM use [30–33]. Only one study focused on understanding the psychological and behavioral factors influencing the use of CAM in cancer patients; however, the study was conducted in Japan, limiting the generalizability of the study findings to other nations [34].

To further understand why cancer patients use CAM, we can view CAM use as a set of health behaviors. Doing so, we can draw upon the years of rigorous research in health behaviors to understand the beliefs and attitudes underlying CAM use. In particular, research has shown that applying a theoretical model will both increase the ability to predict health behaviors as well as lead to development of interventions to change behaviors [35]. A critical step in beginning to incorporate health behavior methodology in CAM research is the development and validation of an instrument that can measure the attitudes and beliefs predictive of CAM use among cancer patients.

 We chose the theory of planned behavior (TPB) [36] as a conceptual framework to guide the development of the instrument. TBP posits that intentions to use CAM are an important precursor of health behaviors and are influenced by factors such as attitudes, subjective norms, and perceived behavioral control. The TBP has been applied in hundreds of health behavior and health service research studies and has been found to be predictive of health behaviors as well as to inform effective behavioral change interventions [37, 38]. We also chose the TPB because it is conceptually simple and may help point out the major constructs that influence CAM use; as such, it can serve as a starting point for further research and inform intervention development to affect appropriate integration of CAM into cancer care.

Thus, this study aims to develop and validate attitudes and beliefs about complementary and alternative medicine (ABCAM), an instrument capable of reliably measuring the behavioral predictors of CAM use among cancer patients. We hypothesize the factor structure of the instrument to be consistent with that of the TPB domains and that the score of the instrument will be reliable and valid.

## 2. Methods

### 2.1. Instrument Development

We developed the items for this instrument through a systematic and critical review of the existing literature on decision making about CAM use in cancer and in the general population to identify relevant conceptual models, instruments, and concepts. Additional items were informed basing on qualitative interviews and modified-grounded theory analysis conducted among 25 breast cancer survivors between 2008 and 2010 [39]. The qualitative interviews were conducted using TPB as a theoretical framework. Informed by our qualitative research and literature search, initial instrument items were drafted to evaluate the specific behavioral predictions of CAM use.

The initial items were reviewed by members (*N* = 27) of the Penn Integrative Oncology Working Group for face validity (March 2010). These members consisted of physicians, nurses/nurse practitioners, psychosocial support staff (e.g., psychologists, social workers, and nutritionists), CAM practitioners (e.g., massage therapists, Reiki practitioners, acupuncturists, and yoga instructors), and patient representatives. The conceptual model and items of the questionnaire were revised based on feedback from the content experts and stakeholders. Next, cognitive interviews were conducted among patients with different types of cancer. Participants were encouraged to share their thoughts about the items with the researcher as they responded to them to provide feedback about the draft instrument, which included content, clarity, and burden. Items were then revised again in discussion with key collaborators (JM, KD, and KA). The initial scale consisted of 25 items (see appendix). Items assessed agreement with statements concerning perceived benefits, barriers, and subjective norms surrounding CAM use on a 5-point Likert scale (“strongly disagree” to “strongly agree”).

### 2.2. Instrument Validation

We administered ABCAM among a convenience sample at three oncology practices of the Abramson Cancer Center of the University of Pennsylvania Health System (Philadelphia, PA, USA) between May and August 2010. Eligible participants were patients aged 18 or older who had a primary diagnosis of cancer and a Karnofsky performance status of 60 or greater (i.e., ambulatory). Additional inclusion criteria included the approval of the patient's oncologist and the patient's ability to understand and provide informed consent in English. Trained research assistants screened medical records and approached potential study subjects in the waiting area of the oncology clinics. After discussing any concerns, and signing the informed consent, each participant was given a self-report survey. The study was approved by the Institutional Review Board of the University of Pennsylvania.

To assess criterion validity, we used the CAM Beliefs Inventory (CAMBI), an instrument developed among British health consumers. The CAMBI is a 15-item scale measuring three aspects of CAM-related treatment beliefs: belief in natural treatment, belief in participation in treatment, and belief in holistic health [40]. A higher score on the CAMBI indicates more positive beliefs about CAM treatments. The subscales had evidence of satisfactory reliability and correlated with CAM use [40]. While the CAMBI was not validated in cancer populations, the constructs have some degree of face validity to cancer patients. We hypothesized that those who hold more holistic views about their health would be likely to have greater expected benefit from CAM therapies; thus offering some evidence of criterion validity.

To measure CAM use, we modified questions from the National Health Interview Survey (NHIS) by asking individuals: “have you used other sources of support or treatment since your cancer diagnosis?” Response options included common CAM items such as natural products (herbs), megavitamins, relaxation techniques (deep breathing and meditation), massage, chiropractic care, acupuncture, yoga, qi gong, and tai chi [7] as well as therapies commonly used in cancer patient populations such as expressive art therapies and energy therapy [9]. Patients answered each option with a dichotomous response (yes; no). We previously used a similar measure in several survey studies and generated the prevalence of CAM use data reflecting that of the national data [41, 42]. Although commonly reported by patients, prayer was not included because findings suggest that factors associated with its use are substantially different from use of nonprayer CAM [7]. Use of any type of CAM was then dichotomized (yes; no).

### 2.3. Analyses

We first performed descriptive analyses to examine missing data and item distribution. We performed a series of principal component factor (PCF) analyses and item reductions to identify the core factor structure of the instrument. The PCF analysis was used because the primary purpose was to identify and compute composite scores for the factors underlying ABCAM. The number of factors was determined by examination of Eigenvalues ≥1.00 and Scree plot [43, 44]. We removed items that cross-loaded greater than 0.3 and retained items that had a loading of 0.5 or greater on the primary factor in an iterative process [45, 46]. Final Varimax-rotated loadings for individual items ranged from 0.5 to 0.9. Oblique rotation was chosen to simplify interpretation of factors, but summation scores rather than factor scores were ultimately examined to avoid overfitting. Cronbach's alpha statistics were calculated to determine the internal consistency of the scale. Coefficients of 0.70 or greater are considered to be acceptable for an instrument developed to evaluate differences in group means [47]. To evaluate construct validity, we used the Student's *t*-test to compare the scores in each domain between CAM users and nonusers. We hypothesize that greater perceived benefit, lesser perceived barriers, and perceived positive subjective norms are associated with CAM use behaviors. To investigate criterion validity, we correlated ABCAM subscales with the CAMBI [40]. It was expected that perceived benefits and social norms would be positively correlated to domains of CAMBI and that perceived barriers would be negatively correlated to the domains in CAMBI. Data analysis was performed using SPSS 19.0 for Windows (IBM SPSS Statistics 19.0). All statistical tests were two-sided with *P* < 0.05 indicating significance. We chose a sample size of at least 300 to allow adequate power to estimate reliability of the instrument [48].

## 3. Results

Among the 317 participants (83% response rate), the mean age was 58.4 with a standard deviation (SD) of 12.1; 244 (77.2%) were Caucasian; 56 (17.7%) were African American; 7 (2.2%) were Asian; 6 (1.9%) were Hispanic; 3 (0.9%) identified themselves as other. While 88 (27.9%) reported an education status of high school or less, 79 (25.1%) had some graduate or professional education. Overall, 103 (32.5%) of the participants were diagnosed with lung cancer, 88 (27.8%) with breast cancer, 79 (24.9%) with gastrointestinal cancer, and 47 (14.8%) with another type of cancer.

### 3.1. Factor Analysis

**Figure 1:**
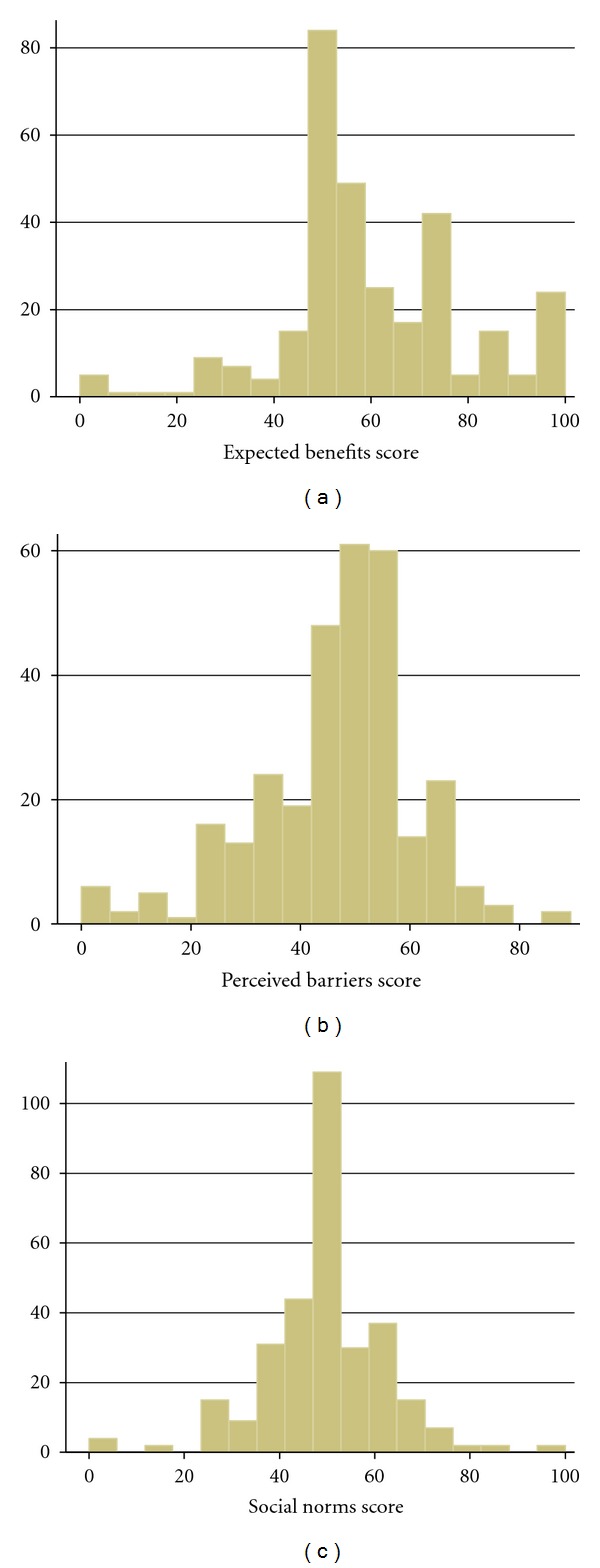
Distribution of domain scores of the ABCAM.

Of the 25 items included in the initial instrument, one item, “reduce stress,” had missing data greater than 5% and was excluded from analysis. The remaining 24 items had missing data ranging from 1.5% to 4.4% with no apparent ceiling or flooring effects. Through iterative factor analysis, we removed items that cross-loaded to multiple domains as well as items that had low correlation coefficients to the intended domains. For example, “boost my immune system,” “my family encourages me to use CAM,” and “my friend asks me to try CAM” cross-loaded to both expected benefits and social norms. Our final scale consisted of 15 items with a 3-factor structure: expected benefits, perceived barriers, and subjective norms (see Table 1). These three domains had Eigenvalues of 4.79, 2.37, and 1.43, and, together, explained over 57.2% of the variance in items. The component scores were then calculated by summing the individual items and normalizing to a value between 0 and 100 for each of the domains (see Table 2 and Figure 1 for distribution of domain scores).

### 3.2. Reliability

The 4-item expected benefits, 7-item perceived barriers, and 4-item subjective norms domain scales each had an acceptable internal consistency (Cronbach's alpha coefficient) of 0.91, 0.76, and 0.75, respectively, (Table 2).

### 3.3. Construct Validity

**Figure 2:**
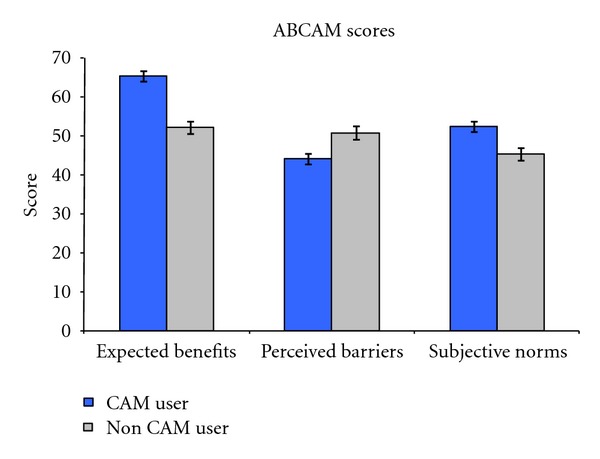
ABCAM domain scores by CAM users versus non-CAM users.

Among the participants, 192 (60.6%) of participants had used at least one type of CAM therapy since cancer diagnosis. The most common approaches were vitamin supplements (120, 34.0%), relaxation techniques (77, 24.4%), herbs (75, 23.8%), special diet (64, 20.5%), and massage therapy (55, 17.4%). As hypothesized, CAM users had higher expected benefits (65.2 versus 52.1, *t* = −5.79, *P* < 0.001), lower perceived barriers (43.9 versus 50.7, *t* = 3.62, *P* < 0.001), and more positive subjective norms (52.3 versus 45.2, *t* = −4.96, *P* < 0.001) associated with CAM than those who did not use CAM (see Figure 2).

### 3.4. Criterion Validity

 To provide a preliminary examination of the ABCAM scale's criterion validity, Pearson's correlations were calculated between ABCAM scale scores and CAMBI scores (see Table 3). The expected benefit score was positively correlated to both preference for natural therapies and a holistic view of health. The perceived barrier score was negatively correlated to belief in participation in treatment decision and holistic health. The positive social norm score was also positively correlated to belief in holistic health. Interestingly, correlations between domain scores in ABCAM and CAMBI were small-to-moderate suggesting that our instrument is measuring different constructs from the CAMBI.

## 4. Discussion

This study sought to develop and validate the ABCAM instrument to measure the decision factors related to the use of CAM among cancer patients. The conceptual model of ABCAM was guided by TPB. It was developed through the literature review, qualitative research, expert review, pilot testing, and quantitative psychometric analysis. The final instrument consists of 15 items measuring three domains related to the attitudes and beliefs predictive of CAM use: expected benefits, perceived barriers, and subjective norms. The scores appear to be reliable and valid in our study population. As hypothesized, CAM users reported higher expected benefits, lower perceived barriers, and more positive subjective norms associated with CAM than those who did not use CAM.

In comparison to existing questionnaires [13, 34, 49–51], the ABCAM is the only one we know that has gone through the process from development to validation in cancer patients. The theoretical model and content of our scale had similarities to the scale developed by Hirai et al., however, the perceived negative outcomes of CAM as measured by Hirai et al. did not include the barriers related to CAM use, which our instrument improves upon. Additionally, all three domains of the ABCAM instrument, including perceived benefits, perceived barriers, and subjective norms, demonstrated higher internal consistency than those reported by Hirai et al. [34].

Our study showed that higher scores of perceived benefits were associated with CAM use among cancer patients. Previous research has shown that cancer patients often use CAM because perceiving it will improve their physical and emotional health, enhance their quality of life, strengthen their immune system, reduce symptoms, and have a positive effect on cancer [10–12, 19–21]. Perceived positive outcomes of CAM use were associated with higher CAM use among a sample of Japanese cancer patients in a prior study [34]. It is important to note that while immune enhancement was a response endorsed by participants, this item cross-loaded to social norm which did not get retained in our final shortened instrument because it did not contribute to the unique factor structure of the instrument. This further suggests the belief that CAM improving one's immune system appears to be socially constructed.

The literature suggests that some of the barriers toward the use of CAM include lack of knowledge, perceived ineffectiveness, cost, time constraint, access to the provider, and perceived side effects of CAM therapies [10, 18, 52–54]. As expected, our study showed that cancer patients who used CAM demonstrated lower perceived barriers as compared to non-CAM users. The domain of perceived barriers represents the construct of perceived behavioral control in the TPB. It is important to note that some of barriers listed are experienced by individuals but they are probably structural barriers (e.g., cost, and access) as well. Therefore, these barriers may be beyond the control of many individuals and will require policy change, insurance coverage, and design of an integrative health care delivery system to ultimately influence change.

Prior studies found that CAM users were more likely to be of female sex, younger age, higher socioeconomic status (e.g., education, and income), and white race [1, 10–13]. Our barrier domain may help understand what specific barriers are experienced among different sociodemographic groups. As evidence accumulates regarding the potential efficacy of some of the CAM therapies in cancer symptom management, this understanding may help reduce the potential disparity in CAM integration. Using our instrument may help quantify the level and significance of these barriers and to guide interventions to target them.

Subjective norms play an important role in patients' intended and actual health behaviors. Patients are more likely to use CAM if it is recommended by their family/friends and/or their health care providers [34, 52]. Our study revealed that CAM users had more positive subjective norms than non-CAM users. This suggests that social approval or disapproval may play an important role in influencing patients' use of CAM therapies; however, our items of family/friend influence cross-loaded between expected benefits and social norm and thus were removed from the final instrument. Consistent with prior qualitative research [25, 55], our data further strengthens the evidence that family/friends' opinions help shape an individual's expected benefit of CAM use; thus, its social normative effect cannot be separated from patients' expected benefits derived from the therapy. Another possible explanation is that cancer patients often consider the opinion of their treating specialist as most important and follow their advice [56–59]. As our instrument is investigated in future research, we can tease out how sources of social influence may shape expectations of therapeutic benefits as well as decisions to use a particular therapy.

The limitations to this study need to be acknowledged. First, our qualitative interviews were conducted with breast cancer patients in the context of decision making about acupuncture; the content of the instrument may not be complete. However, our questionnaire items were also supplemented from the existing literature and then discussed among content experts and patients with other cancers during cognitive interviews. Second, our instrument was guided by TPB as a conceptual framework and well captured the domains in TPB, but like any conceptual model, it may not fully capture other important constructs such as preferences for natural therapies, holistic health view, and finding hope [39, 40, 60]. Additionally, we created a brief instrument that can be incorporated into future cancer epidemiology and health service research; thus, the format of ABCAM is not a traditional TPB instrument. Third, our CAM use was based on self-report and may not capture all of the CAM therapies used by individuals; however, 60.9% use is in the range of what is reported in existing literature [3]. Forth, nonparticipation bias is always a concern in an epidemiology study. Our 83% participation rate is acceptable in survey research, but cannot rule out the potential for selection bias. Lastly, our study was conducted in a large academic cancer center, and future research, including community cancer practices, is needed to increase the generalizability of this study.

In conclusion, this study provided the initial evidence that the ABCAM produced a reliable and valid score for measuring the behavioral predictors of CAM use. Future research is needed to demonstrate additional aspects of reliability and validity (e.g., confirmatory factor analysis; test-retest reliability; sensitivity to change). In addition, prospective research is needed to determine whether these attitudes and beliefs—expected benefits, perceived barriers, and subjective norms—predict both intended and actual use of CAM among cancer patients. Ultimately, this instrument will help elucidate how demographic, socioeconomic, and cultural issues may relate to these attitudes and beliefs, thereby influencing CAM use in the context of cancer care. Such understanding is necessary to guide the appropriate integration of CAM into the conventional health system to improve the health and wellbeing of diverse populations of cancer patients.

## Supplementary Material

Attitudes and Beliefs about Complementary and Alternative Medicine.

## Figures and Tables

**Table 1:** Factor loadings and communalities based on a principal components analysis*.

	Components		
	Expected benefits	Perceived barriers	Social norms
I expect using CAM will decrease my emotional distress	**.88**	−.15	.20
I expect using CAM will reduce symptoms such as pain or fatigue related to cancer and its treatment	**.86**	−.14	.25
I expect using CAM will prevent future development of health problems	**.75**	−.09	.28
I expect using CAM will help me cope with the experience of having cancer	**.91**	−.11	.17
I am unlikely or hesitant about using CAM because it may interfere with the conventional cancer treatment	−.29	**.66**	−.07
I am unlikely or hesitant about using CAM because treatments may have side effects	−.19	**.74**	−.05
I am unlikely or hesitant about using CAM because treatments cost too much money	.05	**.59**	.02
I am unlikely or hesitant about using CAM because it is hard to find good practitioners	.17	**.69**	.02
I am unlikely or hesitant about using CAM because I do not have time to go to CAM treatments	−.14	**.63**	−.11
I am unlikely or hesitant about using CAM because I do not have knowledge about CAM treatments	−.13	**.56**	−.24
I am unlikely or hesitant about using CAM because I do not have transportation to CAM treatments	−.12	**.50**	.05
My health care providers (e.g., doctors, nurses, etc.) encourage me to use CAM	.17	−.10	**.74**
My health care providers (e.g., doctors, nurses, etc.) are open to my use of CAM	.18	−.18	**.76**
Other cancer patients think I should use CAM	.15	−.02	**.77**
My online support group encourages me to try CAM	.23	.09	**.68**

Extraction method: principal component analysis.

Rotation method: Varimax with Kaiser normalization.

*Rotation converged in 5 iterations.

**Table 2:** Descriptive statistics for the ABCAM sub-scales.

	No. of items	*M* (SD)	Skewness	Kurtosis	Cronbach's *α*
Expected benefits	4	60.68 (19.49)	−0.09	3.85	.91

Perceived barriers	7	46.10 (13.43)	−0.74	4.29	.76

Social norms	4	49.58 (14.79)	−0.26	4.46	.75

**Table 3:** Relationship between domains in ABCAM and CAMBI*.

	Natural	Participation	Holistic
Expected benefits	0.23 *P* < 0.001	0.079 *P* = 0.17	0.48 *P* < 0.001

Perceived barriers	0.033 *P* = 0.57	−0.18 *P* = 0.002	−0.28 *P* < 0.001

Social norms	0.10 *P* = 0.077	0.011 *P* = 0.84	0.28 *P* < 0.001

*Pearson's correlation.

## Appendix

See Supplementary Material available online at doi:10.1155/2012/798098.
