# Supplementary material for: Development and Validation of an Instrument for Measuring Attitudes and Beliefs about Complementary and Alternative Medicine (CAM) Use among Cancer Patients
Source: Evid Based Complement Alternat Med. 2012 May 30;2012:798098. doi: 10.1155/2012/798098 (PMC3369496; doi:10.1155/2012/798098)
Supplement: Supplementary file 1 — Attitudes and Beliefs about Complementary and Alternative Medicine. [file 798098.f1.docx]

**Appendix:**

Instrument: Attitudes and Beliefs about Complementary and Alternative Medicine

**Complementary and Alternative Medicine (CAM) therapies are forms of treatment used in addition to or instead of usual medical treatments. These may include dietary supplements, herbal preparation, acupuncture, massage therapy, yoga, reiki, etc. Every cancer patient has his or her own opinions about CAM. We are interested in knowing how your opinion may affect your decision about using such treatments in cancer care. Based on your current situation please choose the option from the following statements that best reflects your views regarding CAM:**

| **I expect using CAM will…** | **Strongly Disagree** | **Disagree** | **Not sure** | **Agree** | **Strongly Agree** |
| --- | --- | --- | --- | --- | --- |
| Improve my physical health |  |  |  |  |  |
| Boost my immune system |  |  |  |  |  |
| Decrease my emotional distress * |  |  |  |  |  |
| Reduce stress |  |  |  |  |  |
| Reduce symptoms such as pain or fatigue related to cancer and its treatments* |  |  |  |  |  |
| Help me live longer |  |  |  |  |  |
| Help cure my cancer |  |  |  |  |  |
| Prevent future development of health problems* |  |  |  |  |  |
| Hel p me cope with the experience of having cancer* |  |  |  |  |  |
| **I am unlikely or hesitant about using CAM because…** |  |  |  |  |  |
| Many treatments are not based on scientific research |  |  |  |  |  |
| May interfere with the conventional cancer treatments* |  |  |  |  |  |
| Treatments may have side effects * |  |  |  |  |  |
| Treatments cost too much money* |  |  |  |  |  |
| It is hard to find good CAM practitioners* |  |  |  |  |  |
| I don’t have time to go to CAM treatments* |  |  |  |  |  |
| I don’t have knowledge about CAM treatments* |  |  |  |  |  |
| Some treatments are against my religious / cultural beliefs |  |  |  |  |  |
| Many treatments are not covered by insurance |  |  |  |  |  |
| I don’t have transportation to CAM treatments* |  |  |  |  |  |

**People around us often can influence our decisions to use different types of treatments. Please select the best option to indicate the extent to which you agree or disagree with the following statements:**

|  | **Strongly disagree** | **Disagree** | **Not sure** | **Agree** | **Strongly agree** |
| --- | --- | --- | --- | --- | --- |
| My family encourages me to use CAM |  |  |  |  |  |
| My health care providers (e.g. doctors, nurses, etc.) encourage me to use CAM* |  |  |  |  |  |
| My health care providers (e.g. doctors, nurses, etc) are open to my use of CAM* |  |  |  |  |  |
| Other cancer patients think I should use CAM* |  |  |  |  |  |
| My online support group encourages me to use CAM* |  |  |  |  |  |
| My friends asks me to try CAM |  |  |  |  |  |

*15 items retained in the final instrument
